# Ovarian reserve markers and assisted reproductive technique (ART) outcomes in women with advanced endometriosis

**DOI:** 10.1186/1477-7827-12-120

**Published:** 2014-12-01

**Authors:** Safiya A Wahd, Shahla K Alalaf, Talha Al-Shawaf, Namir G Al-Tawil

**Affiliations:** Department of Obstetrics and Gynecology, College of Medicine, Hawler Medical University, Erbil, Iraq; Barts and The London Medical College, Women’s Health Research Unit, Centre for Primary Care and Public Health, Queen Mary University, London, UK; Department of Primary Care and Public Health, Imperial College, London, UK; Department of Community Medicine, College of Medicine, Hawler Medical University, Erbil, Iraq

**Keywords:** Stage III-IV endometriosis, Antral follicle count (AFC), Mature oocyte, Intracytoplasmic sperm injection (ICSI), Live birth rate

## Abstract

**Background:**

The role of ovarian reserve markers as predictors of the controlled ovarian stimulation (COS) response in intracytoplasmic sperm injection (ICSI) cycles in women with endometriosis has been much debated. The aim of the present study is to assess the predictability of ovarian reserve markers for the number of mature oocytes (MII) retrieved and to assess the pregnancy rate and live birth rate in women with advanced endometriosis.

**Methods:**

Two hundred eighty-five infertile women who had laparoscopy followed by a first ICSI cycle were recruited in this prospective study. One hundred ten patients were diagnosed with endometriosis stage III-IV (group 1), and 175 patients had no endometriosis (group II). Sixty-three patients in group 1 had no history of previous endometrioma surgery (group Ia), and 47 patients had a history of previous endometrioma surgery (group Ib).

**Results:**

The number of mature oocytes retrieved was significantly lower in women with advanced endometriosis than in women with no endometriosis. The number of mature oocytes retrieved in women with and without endometriosis was best predicted by antral follicle count (AFC) and age, whereas only AFC was a predictor in women with previous endometrioma surgery (odds ratio: 0.49; 95% confidence interval: 0.13-0.60). Women with endometriosis had a lower rate of live births than the control group, but this difference was not statistically significant; the number of live births was significantly lower in those with previous endometrioma surgery.

**Conclusions:**

The best predictor of the COS response in ICSI was AFC, followed by age. Women receiving ICSI following surgery for ovarian endometrioma had a poorer clinical outcome and lower rate of live births compared with those with endometriosis but no previous surgery and the control group.

## Background

Endometriosis is benign but can be a debilitating gynecological disorder and commonly presents with pain and infertility [1]. The exact relationship between endometriosis and infertility remains unclear; surgical findings often do not correlate with fertility potential, nor do they predict therapeutic response [2]. Endometriosis is a common gynecological disorder that occurs in 6-10% of the female population and is more frequent (25-40%) in infertile women [3]. The reasons for this decreased fertility in patients with endometriosis are unclear but may include anatomical distortion, the toxic influence of peritoneal fluid, decreased oocyte or embryo quality, defective endometrial receptivity or diminished ovarian reserve [4]. The use of assessment of ovarian reserve in women with endometriosis undergoing controlled ovarian stimulation (COS) for assisted reproductive technique (ART) has been controversial. Women with endometriosis have been reported to have a lower ovarian reserve and higher basal serum FSH level than their age-matched controls [5, 6]. Other studies suggested that ovarian reserve markers may behave differently in women with and without endometriosis [7, 8]. The adverse effect of endometrioma surgery on ovarian reserve parameters, including serum AMH levels, was recently reported [9, 10]. A few reports in which endometriosis was surgically confirmed reported lower serum anti-Müllerian hormone (AMH) levels in the case of severe endometriosis r-AFS stages III and IV [11]. The presence of endometriosis, particularly ovarian endometrioma, in women undergoing ART has been reported to reduce the response to COS [12, 13]. Al-Azemi et al. demonstrated that despite higher doses of gonadotropins, fewer oocytes were retrieved in ovarian endometriosis cases; this phenomenon was amplified over successive ART attempts [14]. However, not all investigators have found that COS yields were decreased with endometrioma [15, 16]. A meta-analysis of 22 studies reported a lower chance of pregnancy for patients with endometriosis compared to patients with tubal disease (odds ratio: 0.56; 95% CI: 0.44-0.70) [8]. Others have reported no adverse impact of endometriosis on in vitro fertilization (IVF) outcomes compared with other infertility causes [17]. The aim of the present study was to evaluate the predictability of AFC, serum AMH, FSH, and age as predictors of COS response in patients receiving their first ICSI cycle, with laparoscopically diagnosed and histologically confirmed endometriosis with and without endometrioma surgery compared with controls. We further evaluated the impact of endometriosis and previous surgery on clinical outcome.

## Methods

### Study subjects and design

A prospective, cross-sectional study was conducted on 460 infertile women to classify them through laparoscopy according to the presence of endometriosis: those with endometriosis represented the exposed group, and those without endometriosis represented the unexposed group. This study was conducted at the Maternity Teaching Hospital Fertility Centre (MTHFC), Erbil, Kurdistan region, Iraq, from July 1, 2011 to May 1, 2013. All ICSI cycles were performed at MTHFC, which is the only public maternity hospital in the city. The center was established in April 2010 and is the main referral center for infertility in Erbil. Women with a history of infertility are referred to the fertility outpatient clinic, where they are investigated and managed. The standard infertility investigation in our center includes female medical history, clinical examination, gynecological ultrasound, and 2–3 day blood analysis for FSH, LH, estradiol, prolactin, TSH and AMH. Serum progesterone was measured in the midluteal phase of the menstrual cycle for assessment of ovulation. The serum samples were frozen and stored for future analysis. AMH was measured with an ultrasensitive enzyme-linked immunosorbent assay ELISA (AMH Gen II ELISA, Beckman-Coulter, Inc, 250 S. Kraemer Blvd, Brea, CA 92821 U.S.A.). The lowest detection rate limit and intra- and inter-assay variation coefficients were 0.03 ng/mL, 3.4%, and 7.7%, respectively. The unit of measurement for AMH used was ng/mL (1 ng/mL = 7.14 pmol/L). Ovarian ultrasound scanning was performed to evaluate the number and size of antral follicles between 2–10 mm. Antral follicle count involves counting the resting follicles present on both ovaries at the beginning of the proliferative phase of the menstrual cycle by transvaginal ultrasound. All the scans were performed by a single operator on a GE p3 (LOGIG P3, GE Healthcare Wisconsin, USA) equipped with a 6.5 MHz endovaginal probe (probe destination 8CS/E8C). A total of 460 women underwent diagnostic laparoscopy at MTHFC as part of their fertility assessment before starting treatment. At MTHFC, we recommend laparoscopy for all women with 1) infertility for at least 3 years who have regular menstrual cycles (variation 21–35 days) and whose partners have a normal semen analysis, 2) those with abnormal or inconclusive HSG 3) and those who had previous laparoscopy or laparotomy for benign gynecological conditions. All laparoscopies were performed by or in the presence of the first author. When present, the extent of endometriosis was staged surgically according to the American Society for Reproductive Medicine classification of endometriosis [18]. The inclusion criteria of this study were as follows: 1) women receiving their first cycle of ICSI, 2) infertility associated with endometriosis stages III or IV, 3) infertile women with no visual endometriotic lesion as a control group, 4) the absence of polycystic ovarian syndrome (PCOS), and 5) the absence of other endocrine disease (thyroid disease, diabetes mellitus, Cushing’s syndrome). The final study population consisted of 110 women with endometriosis as the study group (group I). The control group (group II) included 175 women undergoing ICSI cycles for infertility not related to endometriosis, The control group were women with tubal factor (47.4%), unexplained infertility (37.7%) and (14.8%) women with mild to moderate male factor infertility. All of them were surgically confirmed for the absence of endometriosis. Women with endometriosis 10% of them had mild to moderate male factor infertility. No severe male factor infertility was found a cross all groups.

Women with endometriosis were further classified according to their surgical history: group Ia with no prior endometrioma surgery (n = 63) and group Ib with a prior history of endometrioma surgery (n = 47). Only women undergoing their first ART cycle were included in the study.

### Ovarian stimulation protocol

COS was conducted according to a down-regulation protocol, which was achieved by subcutaneously (s.c.) administering 0.1 mg triptorelin acetate (Decapeptyl, Ferring Gmbh, D-24109 Kiel, Germany) daily starting from the midluteal phase of the previous menstrual cycle until the day of human chorionic gonadotropin injection. The gonadotropin treatment was commenced from day 2 or 3 of the subsequent cycle if follicular diameters did not exceed 10 mm and the serum estradiol (E2) level was ≤ 50 pg/mL. Patients received a daily starting dosage of 150 to 300 IU of recombinant follicle-stimulating hormone (r-FSH, Gonal-F, Serono Laboratories, Aubonne, Switzerland) s.c. that was individually chosen according to age, body mass index (BMI), basal FSH, and basal AFC. From day 6, the rFSH dose was adjusted according to the ovarian response. When at least two leading follicles reached 18 mm diameter, s.c. injection of 10,000 IU human chorionic gonadotropin (Choriomon, IBSA, Lugano, Switzerland) was administered, and transvaginal ultrasound-guided oocyte retrieval was scheduled for 36 hours later. Intracytoplasmic sperm injection was performed in all cases because the aim of the center was to minimize the incidence of fertilization failure and to maximize the fertilization rate. This policy aided the study in establishing fertilization uniformity and examining the oocytes for maturity. In Erbil and throughout Iraq, IVF/ICSI treatment is not covered by government funding or private insurance; the financial burden has thus forced patients and clinicians to agree to transfer a larger number of embryos than is accepted in many countries. Therefore, the average number of transferred embryos was 3–4. Embryo transfer was performed on day 2 or 3 after oocyte retrieval. Luteal phase support was provided the day after ovum retrieval with a Cyclogest pessary (Actavis, UK) administering 400 mg progesterone daily in addition to 10 mg oral dydrogesterone (Duphaston, Solvay Pharma Istanbul) three times daily. A serum hCG level >20 IU/L was defined as a pregnancy. A transient increase in hCG was defined as a biochemical pregnancy. In all other pregnancies, an ultrasound scan was performed 5–7 weeks after embryo transfer (ET). Clinical pregnancy is defined by ultrasound visualization of an intra-uterine gestational sac and embryonic pole with a heartbeat. All pregnant women were asked to report back, in writing, the outcomes; if women did not report back, they were phoned to inquire about the pregnancy and birth outcome. The primary outcomes were the number of mature oocytes retrieved in the endometriosis patients and the control group, pregnancy rate and live birth rate. The secondary outcomes were the total gonadotropin dose (IU), number of embryos formed and number of good-quality embryos (embryos with blastomers of equal size and no cytoplasmic fragmentation, or < 20% fragmentation).

The Research Ethics Committee of Hawler Medical University, College of Medicine, approved the study proposal on June 24, 2011, (reference number 1/24-06-2011). Informed consent was obtained from all participants after they were fully informed of the aim, process and confidentiality of the study.

### Statistical analysis

Data were analyzed using the Statistical Package for Social Sciences (SPSS, version 19). One-way analysis of variance (ANOVA) was used to compare among means of three or more groups. A post hoc test (LSD) was used to determine whether the difference between two groups was significant (conducted after the ANOVA). A chi square test of association was used to compare between proportions. When the expected count of more than 20% of the cells of the table was less than 5, Fisher’s exact test was used. Multiple regressions were used when the dependent variable was numerical. The power of the study was estimated to be 80% according to the computer program ‘power and sample size estimation’ version 3.0.43. A ‘P’ value of ≤0.05 was considered statistically significant.

## Results

**Figure 1:**
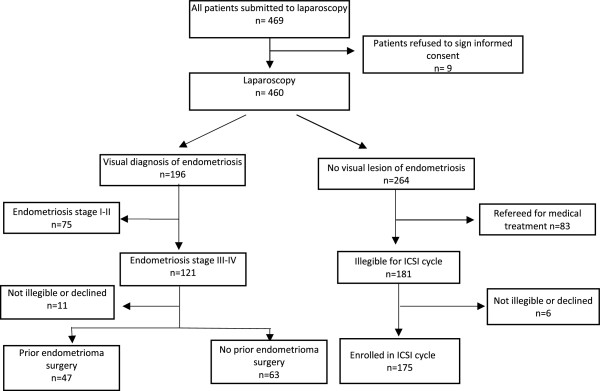
**Patient inclusion flowchart.**

Figure 1: is the total inclusion flow chart.

The mean age of all the studied groups was 32.72 ± 5.76 years. The groups were comparable in age, infertility duration and basal day 3 FSH and estradiol levels (Table 1), but group II had a higher BMI. The AFC was lower in group Ib (9.09 ± 3.07) and group Ia (10.75 ± 3.07) compared with the control group (12.39 ± 4.17) (P < 0.001). The AMH level was lower in group Ib (1.06 ± 0.85) than in group Ia (1.86 ± 1.03, P < 0.003) and group II (2.19 ± 1.57, P < 0.001).

**Table 1:** **Clinical characteristics of women with endometriosis stage III-IV, previous endometrioma surgery and infertility without endometriosis**

Parameters	Stage III and IV endometriosis, n = 110		II-No endometriosis n = 175	Overall P value	P IaXIb	P IaXII	P IbXII
	Ia-No previous surgery, n = 63	Ib-Previous endometrioma surgery, n = 47					
Age (years)	32.67 ± 5.27	32.62 ± 5.63	32.77 ± 5.99	0.983			
Duration (years)	8.62(4.86)	7.32(5.05)	8.32(5.15)	0.381			
AFC	10.75 ± 3.07	9.09 ± 3.07	12.39 ± 4.17	<0.001	0.24	0.003	<0.001
AMH (ng/mL)*	1.86 ± 1.03	1.06 ± 0.85	2.19 ± 1.57	<0.001	0.003	0.103	<0.001
FSH (mIU/mL)	6.61 ± 2.56	6.97 ± 2.75	6.42 ± 2.51	0.422			
E2 (pg/mL)	38.98 ± 17.34	35.93 ± 16.01	40.13 ± 18.44	0.357			
BMI (kg/m^2^)	25.74 ± 4.27	26.25 ± 4.11	29.66 ± 4.86	<0.001	0.571	<0.001	<0.001

AFC: antral follicle count; AMH: Anti-Müllerian hormone; *(Conversion factor to pmol/L X7.14); E2: estradiol; BMI: body mass index.

Groups Ia and Ib had significantly fewer mature oocytes at ovum retrieval than the control group (5.63 ± 2.74, 4.71 ± 2.29 and 6.92 ± 4.47, respectively; P < 0.001) despite requiring a higher dose of gonadotropin (Table 2), whereas the number of embryos developed was similar among the groups. The fertilization rate was lower in group Ib than in the other groups, but this difference was not statistically significant. The pregnancy rate, clinical pregnancy rate and live birth rate per cycle following ICSI treatment in group Ia were 36.5%, 27% and 23.8%, respectively, lower than in the other two groups, but failed to reach statistical significance when compared with the control group (group II), with values of 44.6%, 36.6% and 29.1%, respectively. The live birth rate (8.5%) was significantly lower following ICSI in women who had previous endometrioma surgery (group Ib) than in the control group and group Ia.

**Table 2:** **ART outcomes of women with endometriosis stage III-IV, previous endometrioma surgery and infertility without endometriosis**

Parameters	Stage III and IV endometriosis, n = 110		II-No endometriosis, N = 175	Overall P value	P IaXIb	p IaXII	P IbXII
	Ia-No previous surgery, n = 63	Ib-Previous surgery, n = 47					
Total gonal-F dose, IU	2622 ± 543	2765 ± 552	2307 ± 685	<0.001^a^	0.253	0.001	<0.001
No. of stimulation days	10.98 ± 1.42	10.76 ± 1.54	10.99 ± 1.67	0.663^a^			
Estradiol on day of HCG	1350 ± 849	1182 ± 547	1479 ± 784	0.065^a^			
No. of oocytes retrieved	6.97 ± 3.29	5.96 ± 2.40	8.82 ± 5.13	<0.001^a^	0.245	0.005	0.001
No. of mature oocytes	5.63 ± 2.74	4.71 ± 2.29	6.92 ± 4.47	0.001^a^	0.228	0.027	<0.001
Fertilization rate	0.75 ± 0.18	0.71 ± 0.19	0.73 ± 0.25	0.741^a^			
Embryo no.	4.15 ± 2.08	3.36 ± 1.68	5.08 ± 4.00	0.005^a^	0.234	0.068	0.234
High-quality embryo	3.06 ± 1.42	2.44 ± 1.14	3.31 ± 2.47	0.071^a^			
No. of ET	3.10 ± 1.21	2.79 ± 1.25	3.05 ± 1.10	0.317^a^			
Implantation rate	0.42 ± 0.22	0.37 ± 0.12	0.40 ± 0.16	0.636^a^			
PR/cycle	23/63(36.5)	10/47(21.3)	78/175(44.6)	0.013^b^	0.096	0.300	0.004
Biochemical pregnancy	6/23(26.1)	4/10(40.0)	14/78(17.9)	0.289^b^			
Clinical PR/cycle	17/63(27.0)	6/47(12.8)	64/175(36.6)	0.006^b^	0.097	0.215	0.002
Live birth rate/cycle	15/63(23.8)	4/47(8.5)	51/175(29.1)	0.013^b^	0.043	0.512	0.004
PR/ET	23/60(38.3)	10/44(22.7)	78/170(45.9)	0.019^b^	0.135	0.365	0.006
Clinical PR/ET	17/60(28.3)	6/44(13.6)	64/170(37.6)	0.008^b^	0.096	0.212	0.004
Live birth/ET	15/60(25.0)	4/44(9.1)	51/170(25.5)	0.017^b^	0.043	0.510	0.006
Mmultiple birth/live birth	4/15(26.7)	0/4(0)	11/51(21.6)	0.591^b^			
Cancellation rate	3/63(4.8)	3/47(6.4)	5/175(2.9)	0.557^b^			

^a^Analysis of variance (ANOVA).

^b^Chi-square test.

Multiple linear regression analyses were performed to study the major independent factors (age, AFC, AMH, FSH and estradiol) for the number of mature oocytes retrieved, which was used as the dependent variable (Table 3). The largest influencing independent factor was AFC in women with endometriosis stage III-IV (OR = 0.589, 95% CI = 0.269–0.786). The second most influential independent factor was age (OR = 0.257; 95% CI = 0.006–0.260). In women with prior endometrioma surgery, the only influencing independent factor was AFC (OR = 0.497; 95% CI = 0.134–0.605) and no age influence was found.

**Table 3:** **Multiple linear regression analysis of possible determinants of mature oocytes retrieved in women with stage III and IV endometriosis and control**

	Unstandardized coefficients		Standardized coefficients	T	P	95% Confidence interval for B	
	B	Std. error	B			Lower bound	Upper bound
**a-Factors predicting number of mature oocytes in endometriosis and no previous surgery**							
(Constant)	−1.238	4.480		-.276	0.783	−10.236	7.761
Age (years)	0.133	0.063	0.257	2.103	0.040	0.006	0.260
AFC	0.528	0.129	0.589	4.098	0.000	0.269	0.786
AMH (ng/mL)	0.023	0.361	0.009	0.065	0.948	−0.701	0.748
FSH (mIU/mL)	0.118	0.122	0.103	0.966	0.339	−0.128	0.364
E2 (pg/mL)	−0.013	0.015	−0.083	−0.858	0.395	−0.044	0.018
**b-Factors predicting number of mature oocytes in women with previous endometrioma surgery**							
(Constant)	-.223	2.924		−0.076	0.940	−6.160	5.714
Age (years)	0.049	0.071	0.117	0.684	0.498	−0.096	0.193
AFC	0.369	0.116	0.497	3.182	0.003	0.134	0.605
AMH (ng/mL)	0.246	0.347	0.091	0.709	0.483	−0.458	0.949
FSH (mIU/mL)	0.073	0.101	0.086	0.723	0.474	−0.132	0.279
E2 (pg/mL)	−0.004	0.018	−0.028	−0.222	0.825	−0.040	0.032
**c-Factors predicting number of mature oocytes in women without endometriosis**							
(Constant)	−6.632	2.770		−2.394	0.018	−12.102	−1.161
Age (years)	0.114	0.054	0.151	2.121	0.035	0.008	0.219
AFC	0.643	0.088	0.596	7.341	0.000	0.470	0.816
AMH (ng/mL)	0.147	0.188	0.051	0.781	0.436	−0.224	0.517
FSH (mIU/mL)	0.089	0.096	0.049	0.922	0.358	−0.101	0.279
E2 (pg/mL)	−0.021	0.013	−0.087	−1.672	0.096	−0.046	0.004

Serum AMH, basal FSH and estradiol had no influence on the number of mature oocytes retrieved in either group.

In women with no endometriosis, serum AMH, basal FSH, and estradiol had no influence on the number of mature oocytes retrieved. For the prediction of the number of mature oocytes according to multiple regression analysis, AFC remained the best predictor (OR = 0.596; 95% CI = 0.470–0.816), followed by age (OR = 0.151; 95% CI = 0.008–0.219).

In 9 patients with previous endometrioma surgery a ≤4 cm endometrioma at the time of oocyte retrieval was noted. In 3 women the endometrioma was accidentally punctured at the time of ovum pick up. In one patient after failure of the cycle she developed pelvic abscess.

## Discussion

AMH has gained wide popularity as an ovarian reserve marker because it offers several advantages compared with other markers, it is stable throughout the menstrual cycle and it is relatively independent of hormonal therapy usage [19–21]. In the current study, women with laparoscopically confirmed stage III-IV endometriosis and with a history of endometrioma surgery had a serum AMH level that was significantly lower than in women with no history of surgery for endometrioma and the control group. This finding is in agreement with other studies reporting on the adverse effect of endometrioma surgery on the ovarian reserve [13, 21–25]. Various explanations of the effect of ovarian surgery on the ovarian reserve in endometriosis have been proposed. Bongioanni et al. [26] and Matalliotakis et al. [13] stated that ovarian destruction caused by surgery was the major reason. The lack of a real plane of cleavage between the endometrium-like stroma of the endometriotic cysts and the ovarian tissue can lead to the removal of adjacent healthy ovarian tissue, which may harm the ovarian reserve [27, 28]. However, Kumbak et al. [29] compared ovarian endometriosis and simple ovarian cysts to evaluate the space-occupying effect on the ovary and concluded that the endometriosis itself led to the decline of the ovarian reserve and the poor response. However, some reports showed no differences in ovarian reserve before or after endometrioma resection [30, 31]. In the current study, we observed that the basal serum FSH level was the same between the study groups. Several studies have investigated ovarian reserve in endometriosis. Lemos et al. [32] demonstrated that infertile patients with minimal or mild endometriosis exhibited decreased serum AMH levels compared to a control group. In that study, the two groups’ serum FSH levels did not differ. However, Hock et al. [7] reported a significant increase in basal FSH levels in women with advanced endometriosis, supporting the idea of a progressive loss of ovarian function as the disease advances. Furthermore, Barri et al. [33] found that patients with endometriosis had a higher FSH level and a lower AFC than patients with male-factor infertility before any treatment, especially surgery. AFC has been demonstrated to be a reliable marker of ovarian reserve because it correlates significantly with age-related follicle count decline and with ovarian response to IVF stimulation cycles [19, 20]. In the current study, no significant differences were found in AFC in women with a previous history of endometrioma surgery and no history of endometrioma surgery. However, AFC was significantly lower in both groups than the control group. Our finding is in agreement with the systematic review and meta-analyses performed by Muzii et al. [34], in which the ovarian reserve evaluated with AFC was not reduced after surgical treatment of an endometrioma and a lower AFC was present in both those who had no previous surgery for endometrioma and those who had previous surgery. These data may support the hypothesis that damage to the ovarian tissue is already present before surgery and is therefore caused by the disease itself rather than the surgical procedure [35].

In our study, the response to ovarian stimulation with gonadotropin in terms of the total dose and number of retrieved oocytes differed between patients with ASRM III and IV endometriosis and those with infertility without endometriosis. This finding of an inferior ovarian response in subjects with ASRM III-IV endometriosis confirms prior reports [13, 14, 36, 37]. We further concluded that in women undergoing ICSI with stage III-IV endometriosis with and without a history of endometrioma surgery, despite higher gonadotropin stimulation, the ovarian response was not influenced, as measured by number of oocytes retrieved. In women with extensive endometriosis with and without endometrioma, reports on the outcome of ART, including pregnancy rate and live birth rate, are also conflicting, with some suggesting poor outcomes [38, 39] and others suggesting comparable treatment outcomes [13, 40]. The main finding of our study is that women receiving their first ICSI treatment cycle, specifically those with endometriosis ASRM III-IV, had comparable PRs and live birth rates to women with no endometriosis, whereas the pregnancy rate and live birth rate were significantly lower only in women who had prior endometrioma surgery. Our results are similar to those reported by Opoien et al. [41]. However, a meta-analysis showed that surgery for endometrioma did not have any adverse effects on subsequent responses to stimulation [42]. Furthermore, in a meta-analysis of observational studies, it was concluded that the PR after IVF was similar regardless of endometrioma surgery [43]. Surgery for endometrioma can affect the ovarian response to COS to the point of compromising the ART outcome, even if embryo implantation rates are not affected following ovarian suppression [44, 45]. Poor quality of oocytes has been suggested as one possible cause of infertility in women with endometriosis as a result of higher apoptotic incidence, more alterations of the cell cycle and higher incidence of oxidative stress than in patients with any other infertility cause [46]. In the current study, we demonstrated that only AFC was most significant in predicting the ovarian response in women with previous endometrioma surgery, followed by age. Serum AMH level was not a significant predictor of the ovarian response. This finding concurs with the result of Hock et al. [7], who demonstrated that a progressive loss of ovarian reserve in women with increasing stages of endometriosis was independent of age. However, Yoo et al. [5] reported that the serum AMH represents a more useful ovarian response marker in women with endometriosis than age or FSH, whereas in our study, serum AMH did not predict the ovarian response. Although AFC proved to be a useful predictor of stimulation outcome in IVF, there might be differences in AFC measurements among observers; in our study, the AFC measurement had good reproducibility because there was only one sonographer. Therefore, inter-observer variability did not have an impact on the results of the current study.

The strength of our study lies in the selection of women with endometriosis and the control group based on strict surgical and histological criteria, using clinical data collected prior to laparoscopy and having controls that were all surgically examined to exclude asymptomatic endometriosis. We considered live birth as an endpoint, and all patients were receiving their first ICSI cycle. All laparoscopy was performed by the same team of doctors in the same hospital, and the measurement of AFC was conducted by one sonographer in a single center study. The primary limitation of the study is that in women with previous endometrioma surgery, no information was available regarding the size of the endometrioma and the types of operations were not available or clearly documented. The control group included several types of infertility and this could have influenced the results.

## Conclusions

For women with laparoscopically and histologically confirmed stage III-IV endometriosis receiving their first ICSI cycle, serum AMH levels were significantly low only in women with a previous history of endometrioma surgery, whereas AFC was significantly lower in both groups of endometriosis with and without a previous history of endometrioma surgery compared with the control group. Multivariate analysis confirmed that AFC represents the most useful ovarian response marker to COS in all studied groups, as the number of mature oocytes retrieved increased with increasing AFC but decreased with increasing age. The number of live births that followed the first ICSI cycle, per cycle and per embryo transfer was lowest in those who had previous surgery for endometrioma. Randomized controlled trials will be more effective in establishing whether surgery for endometrioma influences ART outcomes.

## Abbreviations

AMH: Anti-Mullerian hormone
AFC: Antral follicle count
E2: Estradiol
ART: Assissted reproductive outcome
ICSI: Intracytoplasmic sperm injection
CI: Confidence interval
BMI: Body mass index
ET: Embryo transfer.
